# Severe toxicities in amazonian populations and the role of precision medicine in acute lymphoblastic leukemia treatment

**DOI:** 10.1038/s41598-024-80393-3

**Published:** 2024-11-26

**Authors:** Luciana Pereira Colares Leitão, Natasha Monte, Juliana Carla Gomes Rodrigues, Lilian Marques de Freitas, André Maurício Ribeiro-dos-Santos, Ândrea Ribeiro-dos-Santos, Sidney Santos, Sandro José de Souza, Marianne Rodrigues Fernandes, Ney Pereira Carneiro dos Santos

**Affiliations:** 1 Núcleo de Pesquisas em Oncologia, Instituto de Ciências Biológicas, Universidade Federal do Pará, Hospital Universitário João de Barros Barreto, Unidade de Alta Complexidade em Oncologia, Belém, 4487 PA Brazil; 2 Afya Faculdade de Ciências Médicas de Palmas, Palmas 77.017-004, Tocantins, Brazil; 3 Laboratório de Genética Humana e Médica, Instituto de Ciências Biológicas, Universidade Federal do Pará, Belém, 01 PA Brazil; 4 BioME, Universidade Federal do Rio Grande do Norte, Natal, RN Brazil

**Keywords:** Cancer, Molecular medicine

## Abstract

Corticosteroids, such as prednisone or dexamethasone, constitute integral components of antineoplastic regimens for Acute Lymphoblastic Leukemia (ALL) therapy, albeit accompanied by significant adverse effects. The multifactorial nature of interindividual variability in drug response, encompassing genetic polymorphisms, underscores the complexity of pharmacotherapy outcomes. However, pharmacogenetic investigations hitherto have predominantly focused on cohorts of European and North American descent, thus limiting the generalizability of findings to populations with minimal representation. Indigenous populations in Brazil, particularly those inhabiting the Amazon region, exhibit a distinctive genetic heritage, predominantly characterized by Native American ancestry. These populations frequently manifest suboptimal therapeutic responses and elevated mortality rates following ALL treatment. Therefore, delineating the molecular signatures of genes implicated in the corticosteroid pathway within these indigenous cohorts assumes paramount importance. This study identified novel variants within genes associated with the glucocorticoid pathway in indigenous Amazonian populations and conducted comparative analyses of variant frequencies across diverse global populations. The findings underscore the genetic uniqueness of indigenous groups and highlight the potential impact of genetic factors on adverse responses to ALL treatment. Precision medicine approaches tailored to the genetic peculiarities of indigenous populations emerge as imperative strategies for optimizing therapeutic efficacy and mitigating treatment-related toxicities in these communities.

**Supplementary Information:**

The online version contains supplementary material available at 10.1038/s41598-024-80393-3.

## Introduction

Commonly referred to as corticosteroids, such as prednisone or dexamethasone, these pharmacological agents constitute pivotal components of antineoplastic regimens, playing a fundamental role in the therapeutic management of Acute Lymphoblastic Leukemia (ALL). Despite their indispensable contribution to treatment success, this pharmacotherapeutic class is accompanied by adverse effects, including skeletal complications (osteoporosis or osteonecrosis), susceptibility to infections, and perturbations in glucose and lipid metabolism^1^.

The interindividual variability observed in both drug efficacy and safety profiles arises from multifaceted factors. It is well acknowledged that genetic polymorphisms influencing drug absorption or metabolism significantly contribute to this variability^2^. Pharmacogenetic investigations have consistently identified genetic variants associated with varying degrees of toxicity in patients undergoing specific ALL therapies^3,4^. However, these inquiries remain predominantly confined to European and North American cohorts.

The population dimension in pharmacogenetics assumes particular significance, given that intra-population genetic variation within the same ethnic group is generally less pronounced compared to inter-population disparities^5^. Consequently, extrapolating findings from existing studies to populations with limited representation becomes impracticable.

Brazilian indigenous populations, comprising approximately 1.7 million individuals, or 0.83% of the nation’s populace according to the 2022 census by the Brazilian Institute of Geography and Statistics (IBGE), with 44.5% concentrated in the North of Brazil^6^, exhibit the highest proportion of Native American genetic heritage in the country^7^.

Populations characterized by elevated levels of Native American genetic ancestry tend to exhibit suboptimal therapeutic outcomes and heightened mortality rates following exposure to ALL treatments^8,9^. In this context, delineating the molecular profiles of genes implicated in the corticosteroid pathway within populations harboring distinctive genetic ancestries, notably indigenous communities of the Brazilian Amazon, holds promise for elucidating the etiology of these phenomena and advancing our comprehension of the impact of ethnic variations on drug-related toxicity events.

This study aims to investigate the genetic variants within the glucocorticoid pathway in indigenous Amazonian populations, with a particular focus on understanding how these genetic differences may contribute to severe toxicities during ALL treatment. By employing a combination of pharmacogenetic analysis and comparative variant frequency assessments across global populations, this research identifies novel variants and highlights the genetic uniqueness of these populations. The results suggest a strong connection between genetic factors and the adverse responses to ALL treatment in indigenous groups, emphasizing the need for precision medicine approaches tailored to these communities. This research holds significance as it provides a critical step towards optimizing therapeutic outcomes and reducing mortality rates among indigenous populations undergoing ALL treatment.

## Results

### General analysis of genes and variants

The analysis of the 18 selected genes revealed a total of 253 variants, with their characteristics detailed in Table 1. Among these, 226 were identified as single nucleotide variants (SNVs), 134 of which were located within intronic regions. Out of all the variants, 158 were predicted to have a potential functional impact, as suggested by in silico analysis. These were classified as modifiers, corresponding to variants that are “generally non-coding or affecting non-coding genes, where predictions are difficult or there is no evidence of impact,” as described by Cingolani, P., et al. (2010)^10^.

**Table 1:** General characteristics of variants found in the exome of observed indigenous populations.

Characteristics	*N* (%)
**Mutation type**	
SNV	226 (89.3)
INDEL	27 (10.7)
**Region**	
3’UTR	9 (3.6)
5’UTR	7 (2.8)
Exon (CDS)	79 (31.2)
Intragenic	8 (3.2)
Intronic	134 (52.9)
Others	16 (6.3)
**Consequence**	
Intragenic	8 (3.2)
Intronic	134 (52.9)
Next protein	2 (0.8)
Non synonymous coding	38 (15)
Non synonymous coding + splice site region	1 (0.4)
Protein structural interaction locus	5 (1.9)
Splice site region + intron	8 (3.2)
Synonymous coding	40 (15.8)
Stop codon	1 (0.4)
3’UTR prime	9 (3.6)
5’UTR prime	7 (2.8)
**SNPeff Impact**	
High	6 (2.4)
Moderate	39 (15,4)
Modifier	158 (62.4)
Low	50 (19.8 )

SNV: Single Nucleotide Variant; INDEL: Insertion OR Deletion; CDS: Coding Sequence; UTR: Untranslated Region.

## Identification of indigenous-specific variants

To further investigate unique genetic signatures, an analysis was conducted to identify variants specific to the indigenous study populations. Thirteen variants were identified exclusively in these groups and have not been observed in other global populations according to existing databases, including gnomAD (Table 2 presents details). A notable variant in the IND-NLLA group, located at position 5:163473551 of the *HMMR* gene, exhibited a high in silico functional impact, characterized by the introduction of a premature stop codon. Meanwhile, several newly identified variants in the IND-LLA group were predominantly located within the *FGFR4* gene’s intronic regions, with predicted modifying impacts. Additional findings indicated that variants with similar impacts were identified in the *ADH1C* gene within the INDG-LLA group and in the *NOS1* and *CTNNB1* genes within the INDG-NLLA group.

**Table 2:** Characteristics of variants reported only in the indigenous population of the study.

Population	Position	Reference	Variant	Gene	Region detailed	Impact	Frequency
**INDG-LLA**	chr4:99344935	G	A	*ADH1C*	Non synonymous coding	Moderate	0.1
	chr3:41239384	A	G	*CTNNB1*	Intron	Modifier	0.1
	chr5:177093290	G	C	*FGFR4*	Non synonymous coding	Moderate	0.1
	chr5:177097403	G	GC	*FGFR4*	Intron	Modifier	0.1
	chr5:177097408	T	G	*FGFR4*	Intron	Modifier	0.25
	chr5:177097412	A	C	*FGFR4*	Intron	Modifier	0.167
	chr5:177097416	A	C	*FGFR4*	Intron	Modifier	0.1
	chr5:177097419	A	C	*FGFR4*	Intron	Modifier	0.1
	chr5:177097422	T	A	*FGFR4*	Intron	Modifier	0.125
**INDG-NLLA**	chr5:163473551	G	T	*HMMR*	Stop gained	High	0.012
	chr12:117330580	C	A	*NOS1*	Non synonymous coding	Moderate	0.012
	chr3:41224558	C	T	*CTNNB1*	Non synonymous coding	Moderate	0.018
	chr3:41239384	A	G	*CTNNB1*	Intron	Modifier	0.009
	chr20:57265970	C	A	*BMP7*	Synonymous coding	Low	0.009

## Frequency analysis of significant variants

Given the extensive dataset, Table 3 presents results from Fisher’s exact test comparison of variants with significant p-values (< 0.05) across at least three populations and with high or moderate functional impacts. Variants that were significant in fewer than three populations or had low impacts are provided separately in Supplementary Table S1. Corresponding allele frequencies for the variants in Table 3 are available in Supplementary Table S2.

When comparing allele frequencies between the IND-NLLA population and other groups, three variants exhibited significantly different frequencies. For instance, variant rs11068428 (*NOS1*) displayed a notably higher frequency in the IND-NLLA group (0.8243), whereas variants rs2032582 (*ABCB1*) and rs351855 (*FGFR4*) showed lower frequencies in IND-NLLA (0.0119 and 0.0116, respectively) compared to the other six populations. Additionally, the high-impact variant rs1051775 (*GSTA1*) revealed significant frequency differences when comparing the European and South Asian populations, with less pronounced but still notable differences relative to the American population and the IND-LLA group.

**Table 3:** P-values obtained from comparative analysis of variant frequencies between the IND-NLLA Population and other evaluated populations in the study.

Gene	Variant	Impact	IND-NLLA x LLA	IND-NLLA x AFR	IND-NLLA x AMR	IND-NLLA x EAS	IND-NLLA x EUR	IND-NLLA x SAS
*ABCB1*	rs2032582	Moderate	*2.320 × 10* ^*− 35*^ *(-)*	*8.933 × 10* ^*− 51*^ *(-)*	*8.429 × 10* ^*− 22*^ *(-)*	*8.367 × 10* ^*− 18*^ *(-)*	*2.938 × 10* ^*− 22*^ *(-)*	*2.817 × 10* ^*− 12*^ *(-)*
*ADH1C*	rs1693482	Moderate	*1.161 × 10* ^*− 9*^ *(+)*	*6.505 × 10* ^*− 3*^ *(+)*	0.974 *(-)*	*8.127 × 10* ^*− 4*^ *(+)*	*3.072 × 10* ^*− 2*^ *(-)*	0.974 *(-)*
	rs35719513	Moderate	*1.206 × 10* ^*− 6*^ *(+)*	*1.206 × 10* ^*− 6*^ *(+)*	*1.406 × 10* ^*− 2*^ *(+)*	*1.206 × 10* ^*− 6*^ *(+)*	*1.206 × 10* ^*− 6*^ *(+)*	*1.206 × 10* ^*− 6*^ *(+)*
*FGFR4*	rs1966265	Moderate	0.126 *(+)*	*1.211 × 10* ^*− 20*^ *(+)*	*2.313 × 10* ^*− 5*^ *(+)*	0.256 *(+)*	*3.531 × 10* ^*− 9*^ *(+)*	*3.531 × 10* ^*− 9*^ *(+)*
	rs351855	Moderate	*1.540 × 10* ^*− 6*^ *(-)*	*4.025 × 10* ^*− 4*^ *(-)*	*3.699 × 10* ^*− 16*^ *(-)*	*5.577 × 10* ^*− 17*^ *(-)*	*1.718 × 10* ^*− 10*^ *(-)*	*1.202 × 10* ^*− 12*^ *(-)*
	rs376618	Moderate	0.325 *(-)*	*2.413 × 10* ^*− 10*^ *(+)*	*1.016 × 10* ^*− 2*^ *(+)*	0.325 *(-)*	*1.682 × 10* ^*− 6*^ *(+)*	*2.884 × 10* ^*− 2*^ *(+)*
*GSTA1*	rs1051775	High	*5.466 × 10* ^*− 4*^ *(-)*	0.264 *(-)*	*5.466 × 10* ^*− 4*^ *(-)*	0.052 *(-)*	*6.629 × 10* ^*− 12*^ *(-)*	*7.095 × 10* ^*− 9*^ *(-)*
*HMMR*	rs299284	Moderate	*1.835 × 10* ^*− 2*^ *(-)*	*3.135 × 10* ^*− 2*^ *(-)*	0.235 *(-)*	1 *(+)*	*3.221 × 10* ^*− 3*^ *(-)*	*1.835 × 10* ^*− 2*^ *(-)*
	rs299295	Moderate	0.433 *(-)*	*1.191 × 10* ^*− 5*^ *(-)*	*3.376 × 10* ^*− 2*^ *(-)*	1 *(-)*	*2.389 × 10* ^*− 4*^ *(-)*	*7.389 × 10* ^*− 5*^ *(-)*
*NOS3*	rs11068428	Moderate	*2.677 × 10* ^*− 15*^ *(+)*	*6.645 × 10* ^*− 21*^ *(+)*	*1.050 × 10* ^*− 3*^ *(+)*	*2.365 × 10* ^*− 7*^ *(+)*	*2.007 × 10* ^*− 14*^ *(+)*	*3.708 × 10* ^*− 13*^ *(+)*
*PNPLA3*	rs2076213	Moderate	1 *(-)*	*1.064 × 10* ^*− 6*^ *(+)*	*4.548 × 10* ^*− 2*^ *(+)*	*4.937 × 10* ^*− 8*^ *(+)*	*2.218 × 10* ^*− 4*^ *(+)*	*2.218 × 10* ^*− 4*^ *(+)*
	rs2294918	Moderate	*3.279 × 10* ^*− 3*^ *(+)*	0.112 *(+)*	*5.412 × 10* ^*− 3*^ *(+)*	*2.239 × 10* ^*− 2*^ *(+)*	*7.285 × 10* ^*− 10*^ *(+)*	*4.072 × 10* ^*− 5*^ *(+)*
	rs738409	Moderate	0.438 (+)	*2.692 × 10*^*− 16*^ (+)	0.244 (+)	*4.072 × 10*^*− 5*^ (+)	*7.274 × 10*^*− 11*^ (+)	*2.777 × 10–*^*11*^ (+)
*SERPINA6*	rs2228541	Moderate	0.295 (+)	*3.591 × 10*^*− 20*^ (+)	*7.183 × 10*^*− 6*^ (+)	0.874 (+)	*1.903 × 10*^*− 17*^ (+)	*3.490 × 10*^*− 9*^ (+)
*SHMT1*	rs1979277	Moderate	*7.675 × 10* ^*− 3*^ (-)	*1.337 × 10* ^*− 3*^ (-)	*2.504 × 10* ^*− 2*^ (-)	0.112 (+)	*5.049 × 10*^*− 3*^ (-)	1 (-)

## Divergence patterns in genetic profiles (MDS Analysis)

**Fig. 1:**
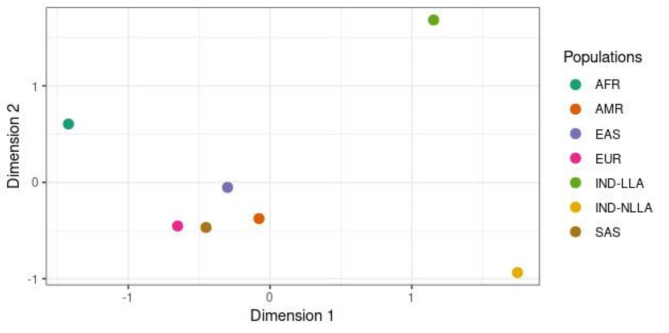
Multidimensional scaling analysis illustrating differences among the genetic profiles of the 8 evaluated populations, considering the frequencies of the 253 variants found in the studied indigenous populations. (African (AFR), American population (AMR), East Asian (EAS), European (EUR), Indigenous individuals with ALL (IND-LLA), Indigenous individuals without ALL (IND-NLLA) and South Asian (SAS)).

To explore genetic divergence patterns among the populations, a Multidimensional Scaling (MDS) analysis was performed based on the criteria outlined in the Methods section. The resulting distribution of points, as depicted in Fig. 1, indicates a marked genetic divergence between the indigenous groups IND-LLA and IND-NLLA and other global populations (AFR, AMR, EAS, EUR, and SAS). Furthermore, the analysis highlights significant genetic distinctions between the IND-LLA and IND-NLLA groups themselves, which can be observed from the distance between the points in the image. The image also suggests that the genetic profiles of both Amazonian indigenous groups align more closely with the American (AMR) populations than with the European (EUR) and African (AFR) populations when considering the 18 selected genes.

## Genetic differentiation among populations (pairwise PERMANOVA analysis)

The results of the pairwise PERMANOVA (Table 4) demonstrated significant differences in genetic composition between the IND-NLLA and IND-LLA populations, with an R² value of 0.058 (F = 3.838, *p* = 0.006). Comparisons between IND-NLLA and continental populations also revealed significant genetic differences when compared with all other populations studied, with the highest R² value observed in the comparison with the African (AFR) population, suggesting that 11.8% of the genetic variation is due to differences between IND-NLLA and AFR (F = 96.375, *p* = 0.006). In contrast, comparisons involving the IND-LLA population with other global populations did not reach statistical significance, except for the comparison with AFR (R² = 0.007, F = 4.913, *p* = 0.055), which approached the threshold for significance. These findings suggest that the genetic profile of the IND-NLLA populations is more distinct from various continental populations compared to that of IND-LLA.

**Table 4:** Pairwise PERMANOVA Analysis of Genetic Variation Between IND-NLLA, IND-LLA, and Global Populations

Populations	*R*²	F value	*p*-value
IND-NLLA vs. IND-LLA	0.058	3.838	0.006
IND-NLLA vs. AFR	0.118	96.375	0.006
IND-NLLA vs. AMR	0.057	24.625	0.006
IND-NLLA vs. EAS	0.093	57.700	0.006
IND-NLLA vs. EUR	0.079	48.208	0.006
IND-NLLA vs. SAS	0.060	34.547	0.006
IND-LLA vs. AFR	0.007	4.913	0.055
IND-LLA vs. AMR	0.003	1.046	0.319
IND-LLA vs. EAS	0.004	2.167	0.309
IND-LLA vs. EUR	0.005	2.322	0.309
IND-LLA vs. SAS	0.003	1.560	0.319

## Discussion

Despite the proliferation of genomic studies in recent decades, our understanding of the genome of specific ethnic groups, such as Amazonian indigenous populations, remains limited. This scarcity of information becomes particularly evident when seeking specific data, such as the genetic component influencing patients’ responses to Acute Lymphoblastic Leukemia (ALL) treatment. Until January 2024, searches conducted in the PubMed database using broad terms like “acute leukemia,” “treatment,” “indigenous populations,” “Native American,” “pharmacogenetics,” and “pharmacogenomics” yielded fewer than 100 studies, many of which were conducted by our research group. In this context, our study stands as the first to investigate genes related to glucocorticoid treatment in ALL within indigenous populations. Our aim is to elucidate the potential causes behind the elevated rates of treatment toxicity observed in this group. We discuss our findings on two fronts: the disparity between the gene profile related to ALL treatment in indigenous populations without the disease compared to large continental populations, and the differences observed in the genetic profiles of the same genes between indigenous individuals with fatal outcomes from ALL treatment and those without the disease.

Among the 13 variants exclusively reported in our study population, distributed across the *CTNNB1*,* ADH1C*,* FGFR4*,* HMMR*, and *BMP7* genes, variants such as chr5:163473551 in *HMMR*, which were predicted to have a high impact on protein activity, and variants chr4:99344935 in *ADH1C* and chr3:41224558 in *CTNNB1*, all non-synonymous variants, were predicted to have a moderate impact. Existing literature provides insights into how alterations in the activity of these gene products could influence the response to ALL treatment.

Fajardo and colleagues^11^ demonstrated the impact of steroid treatment on *HMMR* expression through experiments involving rats with experimentally induced lung inflammation. Steroid use led to a 40% reduction in macrophage accumulation in the lungs of injured animals. This study suggested that steroids decrease *HMMR* expression in vivo and that the anti-inflammatory action in lung injury could be partly explained by gene expression inhibition. In this context, the presence of genetic variants in the *HMMR* gene could potentially affect variation in steroid response and inflammation regulation.

Similarly, Cortese et al.^12^ showed that the glucocorticoid response element of the human *ADH1* gene could be induced by dexamethasone in mice and that it interacts with the glucocorticoid receptor. Dong et al.^13^ demonstrated that dexamethasone treatment caused a dose-dependent increase in total *ADH1* mRNA levels in cells, indicating enhanced transcription. Notably, the *ADH1C* gene, like most alcohol dehydrogenases (ADHs), exhibits peak activity in the liver, a critical organ for drug metabolism in general.

In Shah et al.^14^ study, β-catenin, from the CTNNB1 gene, was linked to dexamethasone resistance. Using a gene signature, they developed a model to predict dexamethasone sensitivity, uncovering pathways leading to resistance. Analysis pinpointed key proteins, including Aurora kinase, S6K, p38, and β-catenin, suggesting their role in dexamethasone resistance, possibly due to the activation of the Aurora kinase/β-catenin axis, contributing to cell survival signaling in resistant patients.

Regarding the results from comparing variant frequencies in the indigenous population with other world populations, only variants rs2032582 (*ABCB1*), rs1966265 and rs351855 (*FGFR4*), rs1051775 (*GSTA1*), rs2294918 and rs738409 (*PNPLA3*), and rs1979277 (*SHMT1*) have been previously evaluated for their impact on therapeutic outcomes.

The rs2032582 (*ABCB1*) variant, with a frequency of 0.012 in the IND-NLLA population and 0.75 in the IND-LLA population, in homozygosity, is associated with decreased overall survival in multiple myeloma patients treated with dexamethasone, according to clinical data available on the PharmGKB platform (https://www.pharmgkb.org*).*

The rs1966265 (*FGFR4*) variant was linked to drug response specifically in cyclophosphamide-epirubicin-docetaxel-based therapy in breast cancer patients, where it was identified as a potential marker for predicting treatment response^15^. The rs351855 (*FGFR4*) variant, in conjunction with a variant in the *NOS3* gene not found in the indigenous population of our study, may serve as a prognostic marker for patients with hepatocellular carcinoma treated with lenvatinib^16^. This variant has also been associated with febrile neutropenia occurrence in breast cancer patients undergoing chemotherapy^17,18^.

The high-impact variant rs1051775 (*GSTA1*) has been linked to mortality related to cyclophosphamide treatment in individuals undergoing hematopoietic stem cell transplantation. The same study suggests that this variant is associated with reduced protein levels, resulting in impaired drug metabolism, potentially leading to increased cellular toxicity; however, further confirmation is needed^19^.

The rs2294918 (*PNPLA3*) variant, with high prevalence in both the IND-LLA and IND-NLLA groups (0.8 and 0.941, respectively), does not affect the resulting protein’s activity, but carriers of the wild-type allele exhibited decreased mRNA levels in the liver^20^. The rs738409 (*PNPLA3*) variant was studied in relation to its association with liver damage in a multi-ethnic cohort of pediatric ALL patients undergoing therapy, where it was found to be associated with altered liver function, as evidenced by increased alanine aminotransferase (ALT) and aspartate aminotransferase (AST) levels^21^. Liu et al.^22^ also associated this variant with elevated ALT levels in children undergoing treatment for B-cell ALL, suggesting a potential association with hepatotoxicity during disease treatment.

The homozygous mutant genotype of the rs1979277 (*SHMT1*) variant was indicated to be associated with an increased risk of toxicity in a study assessing fluoropyrimidine and platinum-based therapy-induced toxicity in gastric cancer patients^23^.

Although other variants lack studies linking them to toxicities and treatment responses, their potential influence on toxicity occurrence in indigenous individuals undergoing ALL treatment should not be disregarded. Another significant variable to consider is the substantial Native American genetic contribution in this population, as highlighted by De Carvalho and colleagues^8^.

The presence of germline genetic variants capable of influencing drug response and associated with Native American ancestry could explain this scenario^8^, as this ancestral group has also been identified as a potential risk factor for standard ALL treatment toxicity in another population: Hispanic individuals exhibit poorer outcomes compared to Caucasians in this regard^24^.

Our findings revealed that 14 variants with moderate impact and 1 with high impact exhibit statistically significant frequency differences across at least 3 of the 5 global populations evaluated. These disparities are corroborated by the multidimensional scaling plot generated from the dissimilarity matrix of the evaluated variants, indicating that the Amazonian indigenous groups studied are more closely related to individuals from the AMR group, who also share genetic ancestry with Native Americans and East Asian individuals^25,26^. Although the pairwise PERMANOVA results showed a statistically significant difference between IND-NLLA and AMR, this was the lowest divergence among the compared groups (excluding IND-LLA), with a genetic divergence of 5.7%. The multidimensional scaling plot also reveals an isolated cluster formed by the African population, consistent with human evolutionary history^27^, which was further corroborated by the pairwise PERMANOVA results.

This allows us to reinforce two propositions previously discussed: (i) the distinct genetic profile of these indigenous groups concerning the variants analyzed in this study, and (ii) the potential role of genetics in the adverse response to ALL treatment in indigenous populations.

The oversight of ethnic-genetic characteristics during the therapeutic process for indigenous populations has resulted in significant consequences, notably evidenced by the elevated incidences of treatment-related toxicities. This phenomenon underscores the urgency of further studies on the genetic profile of indigenous populations. By comprehending the genetic peculiarities of these communities, clinical strategies can be enhanced, enabling personalized treatments tailored in a more effective and adapted manner. Such targeted approaches not only aim to minimize toxicity risks but also represent a pivotal step toward substantially increasing survival rates in these communities, fostering a more inclusive and responsive approach to their unique healthcare needs.

In conclusion, our study identified thirteen novel variants within the 18 genes associated with the glucocorticoid pathway in the indigenous groups under evaluation, notably including a high-impact variant within the HMMR gene. Furthermore, our comparative analysis of variant frequencies between the study groups revealed that out of the 117 variants assessed, 14 with moderate impact and 1 with high impact (rs1051775 in GSTA1) exhibited statistically significant differences across at least 3 of the 6 populations analyzed. It is important to acknowledge, however, that the disparity in sample sizes between the IND-ALL and IND-NALL populations and the other continental populations evaluated, particularly the smaller size of the IND-ALL group, may affect allele frequency comparisons, even though we standardized the frequencies according to the size of the IND-NLLA population to minimize the effect of these differences. These sample size limitations should, therefore, be taken into consideration when interpreting the results.

To deepen our understanding of how genetic variants can impact glucocorticoid response in ALL therapy, further functional and association studies are imperative. However, investigating populations characterized by a high incidence of severe and fatal toxicities, such as indigenous populations, is crucial for directing efforts towards the prevention and management of these adverse events. This underscores the importance of implementing precision medicine strategies tailored to the unique genetic characteristics of these groups.

## Methods

### Ethical compliance

This study obtained approval from the National Research Ethics Commission (CONEP) under protocols 1062/2006 and 123/98. Indigenous participants received a comprehensive explanation of the research design and its significance, with assistance from translators as necessary. Informed consent forms were signed by all participants and their respective leaders. Collection of materials adhered to the principles outlined in the Declaration of Helsinki.

### Samples studied

**Fig. 2:**
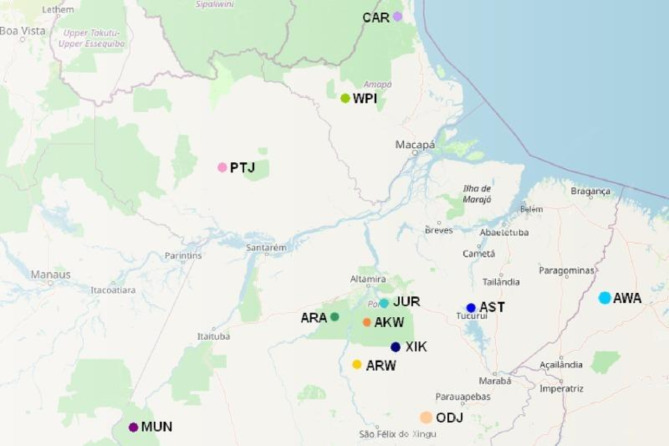
Location of the 13 ethnic groups from the Amazon region of Northern Brazil evaluated in the study. *Asurini do Koatinemo (AKW)*,* Arara/Arara do Iriri (ARA)*,* Araweté (ARW)*,* Asurini do Trocará (AST)*,* Awa-Guajá (AWA)*,* Caripuna (CAR)*,* Juruna (JUR)*,* Munduruku (MUN)*,* Xikrin Odjá (ODJ)*,* Zo’é (PTJ)*,* Wayãpy (WPI)*,* Xikrin do Cateté (XIK).*

Participant recruitment occurred from September 2017 to December 2018. The study cohort comprised Amerindians from the Amazon region of Northern Brazil, categorized into two groups based on their health status: the IND-LLA group (indigenous individuals with Acute Lymphoblastic Leukemia [ALL]), consisting of 5 individuals with the disease exhibiting varying toxicities during treatment, and the IND-NLLA group (indigenous individuals without ALL), comprising 59 disease-free individuals. Ancestry analysis was conducted as described by Ramos et al. (2016)^28^, using 61 autosomal ancestry informative markers (AIMs). Three multiplex PCR reactions were performed with insertion/deletion (INDEL) markers, and the PCR amplifications were analyzed through electrophoresis using the ABI Prism 3130 sequencer and GeneMapper ID v.3.2 software. The individual proportions of European, African, and Native American genetic ancestries were estimated using STRUCTURE v.2.3.3 software, assuming three parental populations (European, African, and Native American). The study enrolled 64 unrelated individuals representing 12 distinct Amazonian ethnicities (5 Assurini do Koatinemo, 7 Arara/Arara do Iriri, 6 Araweté, 16 Asurini do Trocará, 7 Awa-Guajá, 1 Kayapó, 2 Xikrin Odjá, 5 Zo’é, 5 Wayãpy, 6 Xikrin do Cateté, 2 Caripunas, and 2 Jurunas). The geographic distribution of these ethnicities in the Amazonian territory is depicted in Fig. 2. Further details regarding this population can be found in Rodrigues et al.^29^.

For comparison with populations from other continents, we used data obtained from the 1000 Genomes, phase 3 release (available at http://www.1000genomes.org*)*, composed of 661 individuals from Africa (AFR), 503 from Europe (EUR), 347 from the Americas (AMR), 504 from East Asia (EAS), and 489 from South Asia (SAS).

### DNA extraction and Exome Library Preparation

DNA extraction and exome library preparation procedures initially encompassed 58 available samples, as outlined by Ribeiro-Dos-Santos et al.^30^. Subsequently, six additional samples were included, with their respective extractions and exome library preparation described by Rodrigues et al.^29^.

### Gene selection

Gene selection was based on a comprehensive literature search conducted in the PubMed database (pubmed.ncbi.nlm.nih.gov), focusing on high-impact studies from the last 10 years discussing genetic aspects of ALL treatment, specifically related to glucocorticoid therapy. This process identified 18 genes (*ABCB1*,* ACP1*,* ADH1C*,* BCL2L11*,* BMP7*,* CTNNB1*,* CXCL12*,* DROSHA*,* FGFR4*,* FOLH1*,* GATA3*,* GSTA1*,* HMMR*,* NOS1*,* NR3C1*,* PNPLA3*,* SERPINA6*, and *SHMT1*) with known roles in glucocorticoid pharmacological pathways. The selected genes are documented in a Supplementary Table S3, detailing their functions, involved pathways, and supporting references.

### Bioinformatic analyses

Bioinformatic analyses followed methods detailed by Rodrigues et al.^29^ and Ribeiro-Dos-Santos et al.^30^. Initially, sequences underwent filtering to eliminate low-quality reads, followed by alignment to the reference genome (GRCh38) using BWA (v. 0.7). Subsequent processing involved removing duplicate sequences, recalibrating mapping quality, and finalizing local realignment. GATK (v. 3.2) was employed to identify variants relative to the reference genome. Variant annotations were analyzed using the Variant Viewer (ViVa^®^) software, supported by three databases: (1) SnpEff (v. 4.3.T), (2) Ensembl Variant Effect Predictor (Ensembl version 99), and (3) ClinVar (v.2018-10). In silico pathogenicity prediction utilized the following databases: SIFT (v.6.2.1), PolyPhen-2 (v. 2.2), LRT (November 2009), Mutation Evaluator (v. 3.0), Mutation Provider (v. 2.0), FATHMM (v. 2.3), PROVEAN (v. 1.1.3), MetaSVM (v. 1.0), M-CAP (v. 1.4), and FATHMM-MKL.

### Selection of variants

Variant calling identified a total of 253 variants across the 18 analyzed genes (section *Gene Selection*). Subsequent analyses focused on variants meeting specific criteria: a minimum coverage of 10 reads (fastx_tools v.0.13 - http://hannonlab.cshl.edu/fastx_toolkit/*)*, presence in at least 10 of the 64 indigenous individuals, and/or absence in any previously reported populations. Consequently, out of the initial 253 variants, analysis concentrated on the 131 variants meeting the selection criteria.

### Statistical analyses

Allele frequencies in the IND-LLA and IND-NLLA populations were determined by allele counting. Comparisons between the IND-NLLA population and both the IND-LLA group and large continental populations (African [AFR], American [AMR], East Asian [EAS], European [EUR], and South Asian [SAS]) were conducted using Fisher’s exact test with Hochberg correction for multiple comparisons. To account for population size differences, variant frequencies were standardized by adjusting each SNP’s allele count to the IND-NLLA sample size (59 individuals, 118 alleles). The number of ‘yes’ alleles (those with the variant) was calculated as the allele frequency (AF) multiplied by 118, while ‘no’ alleles (those without the variant) were calculated as (1 - AF) multiplied by 118. This standardization facilitated consistent comparisons across populations. To provide a complementary analysis that enhances the understanding of genetic divergence patterns, a pairwise PERMANOVA was conducted to assess the statistical significance of differences in genetic composition both among the INDG populations and in comparison to other global populations, with multiple analyses corrected using the Hochberg method. Significance was set at a p-value ≤ 0.05. Genetic variant allele frequencies were also utilized to compute dissimilarity matrices and construct multidimensional scaling (MDS) plots. All analyses were performed using RStudio v.3.5.1.

## Electronic supplementary material

Below is the link to the electronic supplementary material.

Supplementary Material 1

## Data Availability

The datasets used and/or analysed during the current study available from the corresponding author on reasonable request and all data generated or analysed during this study are included in this published article and its supplementary information files.
