# Supplementary material for: Severe toxicities in amazonian populations and the role of precision medicine in acute lymphoblastic leukemia treatment
Source: Sci Rep. 2024 Nov 26;14:29344. doi: 10.1038/s41598-024-80393-3 (PMC11599904; doi:10.1038/s41598-024-80393-3)
Supplement: Supplementary file 1 — Supplementary Material 1 [file 41598_2024_80393_MOESM1_ESM.docx]

**SUPPLEMENTARY TABLE S1**- P-values from Comparative Analysis of Variant Frequencies Between the IND-NLLA Population and Other Evaluated Populations in the Study, with results significance fewer than three populations.

| **Gene** | **Variant** | **Impact** | **IND-NLLA x LLA** | **IND-NLLA**  **x AFR** | **IND-NLLA**  **x AMR** | **IND-NLLA**  **x EAS** | **IND-NLLA**  **x EUR** | **IND-NLLA**  **x SAS** |
| --- | --- | --- | --- | --- | --- | --- | --- | --- |
| *ABCB1* | rs1045642 | LOW | *1.805 x 10^-06^* | *2.616 x 10^-10^* | *0.016* | *3.562 x 10^-04^* | 0.204 | 0.878 |
|  | rs1128503 | LOW | *8.784 x 10^-15^* | *3.849 x 10^-14^* | *0.002* | 1 | *0.003* | 0.684 |
|  | rs11975994 | MODIFIER | *0.047* | *2.362 x 10^-38^* | *1.021 x 10^-19^* | *3.490 x 10^-09^* | *8.975 x 10^-19^* | *3.584 x 10^-10^* |
|  | rs2235015 | MODIFIER | *1.032 x 10^-05^* | *1.383 x 10^-11^* | *0.003* | 0.365 | *4.013 x 10^-05^* | *2.852 x 10^-04^* |
|  | rs2235047 | MODIFIER | *1.255 x 10^-07^* | *2.722 x 10^-07^* | *8.127 x 10^-04^* | *3.411 x 10^-17^* | 0.581 | *4.057 x 10^-04^* |
|  | rs4148734 | MODIFIER | *1.998 x 10^-13^* | 1 | *1.594 x10^-10^* | *2.852 x 10^-09^* | *3.490 x 10^-09^* | *5.391 x 10^-04^* |
|  | rs4728699 | MODIFIER | 1 | *1.189 x 10^-67^* | *2.282 x 10^-62^* | *1.189 x 10^-67^* | *2.654 x 10^-58^* | *3.159 x 10^-57^* |
| *ACP1* | rs11553746 | MODIFIER | *5.880 x 10^-09^* | 0.061 | *3.452 x 10^-04^* | *0.002* | *4.072 x 10^-07^* | *1.032 x 10^-05^* |
|  | rs12714402 | MODIFIER | *6.871 x 10^-24^* | *1.948 x 10^-21^* | *2.937 x 10^-18^* | *1.508 x 10^-04^* | *7.377 x 10^-10^* | *8.671 x 10^-16^* |
|  | rs144670341 | MODIFIER | *1.161 x 10^-09^* | *1.116 x 10^-05^* | *0.013* | 0.325 | *0.013* | 0.088 |
|  | rs6755722 | MODIFIER | *0.043* | 0.062 | *0.001* | *4.627 x 10^-15^* | *9.758 x 10^-06^* | *8.127 x 10^-04^* |
| ADH1C | rs1693426 | LOW | *1.094 x 10^-10^* | *0.002* | 1 | *1.610 x 10^-04^* | 0.082 | 1 |
|  | rs1612735 | MODIFIER | *6.318 x 10^-04^* | *5.530 x 10^-23^* | *6.350 x 10^-12^* | *1.799 x 10^-25^* | *1.488 x 10^-06^* | *1.546 x 10^-11^* |
|  | rs17526590 | MODIFIER | *1.255 x 10^-07^* | 0.325 | *5.374 x 10^-06^* | *0.024* | *8.127 x 10^-04^* | *8.127 x 10^-04^* |
| *BCL2L11* | rs724710 | LOW | 0.126 | *5.839 x 10^-10^* | 0.684 | *1.007 x 10^-13^* | *2.313 x 10^-05^* | *0.022* |
|  | rs4849417 | MODIFIER | *0.007* | *1.473 x 10^-12^* | 0.502 | *2.267 x 10^-09^* | *3.160 x 10^-04^* | 0.804 |
| *CTNNB1* | rs2293303 | LOW | 0.185 | *1.206 x 10^-06^* | 1 | 0.576 | *1.206 x 10^-06^* | *1.206 x 10^-06^* |
|  | rs2293303 | LOW | 0.185 | *1.206 x 10^-06^* | 1 | 0.576 | *1.206 x 10^-06^* | *1.206 x 10^-06^* |
|  | rs11564465 | MODIFIER | *8.127 x 10^-04^* | *0.003* | *4.061 x 10^-12^* | *5.719 x 10^-09^* | *3.411 x 10^-17^* | *8.784 x 10^-15^* |
|  | rs11564465 | MODIFIER | *8.127 x 10^-04^* | *0.003* | *4.061 x 10^-12^* | *5.719 x 10^-09^* | *3.411 x 10^-17^* | *8.784 x 10^-15^* |
| *CXCL12* | rs197452 | MODIFIER | *1.256 x 10^-07^* | *8.127 x 10^-04^* | *2.037 x 10^-04^* | *0.001* | *5.374 x 10^-06^* | *2.329 x 10^-05^* |
|  | rs2297630 | MODIFIER | *6.448 x 10^-06^* | 0.426 | *7.274 x 10^-11^* | 0.164 | *7.389 x 10^-05^* | *0.003* |
|  | rs266088 | MODIFIER | *1.032 x 10^-05^* | *0.002* | *3.416 x 10^-08^* | *9.840 x 10^-04^* | *9.840 x 10^-04^* | *7.704 x 10^-05^* |
|  | rs197452 | MODIFIER | *1.255 x 10^-07^* | *8.127 x 10^-04^* | *2.037 x 10^-04^* | *0.002* | *5.374 x 10^-06^* | *2.329 x 10^-05^* |
|  | rs2297630 | MODIFIER | *6.448 x 10^-06^* | 0.426 | *7.274 x 10^-11^* | 0.164 | *7.389 x 10^-05^* | *0.003* |
|  | rs266088 | MODIFIER | *1.032 x 10^-05^* | *0.002* | *3.416 x 10^-04^* | *9.840 x 10^-04^* | *9.840 x 10^-04^* | *7.704 x 10^-05^* |
| *DROSHA* | rs2287584 | LOW | *1.805 x 10^-06^* | *0.001* | 0.426 | *6.113 x 10^-08^* | 0.475 | 0.234 |
|  | rs2287584 | LOW | *1.805 x 10^-06^* | *0.001* | 0.426 | *6.113 x 10^-08^* | 0.475 | 0.234 |
|  | rs10052174 | MODIFIER | *3.618 x 10^-04^* | *2.599 x 10^-05^* | 0.827 | *1.475 x 10^-05^* | 0.340 | 0.071 |
|  | rs10719 | MODIFIER | *0.003* | *3.268 x 10^-06^* | 0.501 | *2.825 x 10^-08^* | 0.237 | 0.288 |
|  | rs13183642 | MODIFIER | *1.255 x 10^-07^* | *9.872 x 10^-05^* | *5.365 x 10^-10^* | *8.784 x 10^-15^* | *2.415 x 10^-10^* | *2.124 x 10^-11^* |
|  | rs16901229 | MODIFIER | *0.014* | *0.006* | 1 | *0.009* | 0.189 | 0.088 |
|  | rs3763075 | MODIFIER | *2.329 x 10^-05^* | *0.005* | 0.119 | *1.321 x 10^-12^* | 0.515 | 0.581 |
|  | rs6886834 | MODIFIER | 0.060 | *1.471 x 10^-11^* | 0.060 | *7.278 x 10^-13^* | *0.012* | *0.007* |
|  | rs72552345 | MODIFIER | *1.797 x 10^-04^* | *3.280 x 10^-04^* | 0.332 | *5.086 x 10^-05^* | 0.473 | 1 |
|  | rs10052174 | MODIFIER | *3.618 x 10^-04^* | *2.599 x 10^-05^* | 0.827 | *1.475 x 10^-05^* | 0.340 | 0.071 |
|  | rs10719 | MODIFIER | *0.003* | *3.268 x 10^-06^* | 0.501 | *2.825 x 10^-08^* | 0.237 | 0.288 |
|  | rs13183642 | MODIFIER | *1.255 x 10^-07^* | *9.872 x 10^-05^* | *5.365 x 10^-10^* | *8.784 x 10^-15^* | *2.415 x 10^-10^* | *2.124* *x 10^-11^* |
|  | rs16901229 | MODIFIER | *0.014* | *0.006* | 1 | *0.009* | 0.189 | 0.088 |
|  | rs3763075 | MODIFIER | *2.329 x 10^-05^* | *0.005* | 0.119 | *1.321 x 10^-12^* | 0.515 | 0.581 |
|  | rs6886834 | MODIFIER | 0.060 | *1.471 x 10^-11^* | 0.060 | *7.278 x 10^-13^* | *0.012* | *0.007* |
|  | rs72552345 | MODIFIER | *1.797 x 10^-04^* | *3.280 x 10^-04^* | 0.332 | *5.086 x 10^-05^* | 0.473 | 1 |
|  | rs7735863 | MODIFIER | *0.002* | *5.816 x 10^-09^* | 0.465 | *0.018* | *0.003* | *1.610 x 10^-04^* |
| *FGFR4* | rs446382 | LOW | 1 | *1.459 x 10^-15^* | *2.329 x 10^-05^* | 0.325 | *4.903 x 10^-11^* | *8.127 x 10^-04^* |
|  | rs452885 | LOW | 0.325 | *2.415 x 10^-10^* | *0.010* | 0.325 | *1.682 x 10^-06^* | *0.029* |
|  | rs31776 | LOW | 0.581 | *4.762 x 10^-13^* | *4.013 x 10^-05^* | 1 | *1.603 x 10^-09^* | *0.031* |
|  | rs387598 | MODIFIER | *3.159 x 10^-57^* | *1.396 x 10^-08^* | *0.022* | *0.024* | *7.389 x 10^-05^* | 0.563 |
|  | rs422421 | MODIFIER | 1 | *3.905 x 10^-08^* | *0.003* | 1 | *7.643 x 10^-07^* | *0.031* |
|  | rs434434 | MODIFIER | 1 | *1.161 x 10^-09^* | *1.116 x 10^-05^* | 1 | *1.228 x 10^-08^* | *0.024* |
|  | rs442856 | MODIFIER | 1 | *1.786 x 10^-08^* | *1.053 x 10^-04^* | 1 | *1.726 x 10^-07^* | 0.096 |
| *FOLH1* | rs175849 | MODIFIER | *0.031* | 0.129 | *0.007* | 0.088 | *0.001* | 0.373 |
|  | rs4315497 | MODIFIER | *7.784 x 10^-19^* | *3.582 x 10^-05^* | *0.042* | 0.099 | 0.077 | 0.459 |
| *GATA3* | rs422628 | MODIFIER | *8.671 x 10^-07^* | *4.197 x 10^-07^* | *0.029* | 0.581 | *6.702 x 10^-06^* | *5.918 x 10^-04^* |
| *HMMR* | rs17061707 | MODIFIER | *1.255 x 10^-07^* | 0.581 | *0.024* | *0.047* | 0.088 | 0.581 |
|  | rs184181 | MODIFIER | *9.872 x 10^-05^* | *2.415 x 10^-10^* | *5.374 x 10^-06^* | *0.013* | *2.620 x 10^-09^* | *5.365 x 10^-10^* |
|  | rs2287777 | MODIFIER | *7.278 x 10^-13^* | *2.329 x 10^-05^* | *0.047* | 0.088 | 0.088 | 0.581 |
|  | rs2303076 | MODIFIER | *1.255 x 10^-07^* | *8.127 x 10^-04^* | *1.228 x 10^-08^* | *2.415 x 10^-10^* | *5.719 x 10^-09^* | *5.327 x 10^-18^* |
| *NOS1* | rs1047735 | LOW | *2.721 x 10^-14^* | *2.675 x 10^-20^* | *0.003* | *1.206 x 10^-06^* | *7.560 x 10^-14^* | *1.998 x 10^-13^* |
|  | rs2293054 | LOW | *5.779 x 10^-11^* | *1.857 x 10^-28^* | *1.329 x 10^-04^* | *8.010 x 10^-40^* | *2.692 x 10^-16^* | *2.957 x 10^-17^* |
|  | rs11282297 | MODIFIER | 0.313 | *5.820 x 10^-40^* | *5.820 x 10^-40^* | *5.820 x 10^-40^* | *** | *5.820 x 10^-40^* |
|  | rs12811583 | MODIFIER | *0.047* | *0.029* | *2.528 x 10^-05^* | *1.045 x 10^-07^* | *0.029* | *2.078 x 10^-07^* |
|  | rs2291908 | MODIFIER | *9.322 x 10^-11^* | *3.985 x 10^-16^* | 0.079 | *3.945 x 10^-04^* | *2.250 x 10^-04^* | *1.073 x 10^-07^* |
|  | rs816345 | MODIFIER | *2.947 x 10^-15^* | *6.944 x 10^-35^* | *6.749 x 10^-18^* | *1.919 x 10^-17^* | *1.971 x 10^-08^* | *1.068 x 10^-09^* |
|  | rs816363 | MODIFIER | *3.775 x 10^-07^* | *0.012* | *0.002* | *5.067 x 10^-08^* | *6.140 x 10^-10^* | *3.775 x 10^-07^* |
|  | rs9658354 | MODIFIER | *6.311 x 10^-04^* | *0.001* | *9.593 x 10^-12^* | *4.055 x 10^-08^* | *3.090 x 10^-07^* | *4.930 x 10^-11^* |
|  | rs9658463 | MODIFIER | *1.255 x 10^-07^* | *5.867 x 10^-22^* | *0.024* | *0.047* | 1 | 0.581 |
| *NR3C1* | rs4986593 | MODIFIER | *1.032 x 10^-05^* | 1 | *1.508 x 10^-04^* | *4.013 x 10^-05^* | *6.341 x 10^-07^* | *4.013 x 10^-05^* |
| *PNPLA3* | rs738408 | LOW | 0.438 | *2.692 x 10^-16^* | 0.244 | *4.072 x 10^-05^* | *7.274 x 10^-11^* | *2.777 x 10^-11^* |
|  | rs139047 | MODIFIER | *1.255 x 10^-07^* | *1.228 x 10^-08^* | *5.719 x 10^-09^* | *5.327 x 10^-18^* | *5.327 x 10^-18^* | *7.278 x 10^-13^* |
|  | rs139051 | MODIFIER | 1 | *5.757 x 10^-09^* | 0.082 | *0.016* | *2.616 x 10^-10^* | *3.839 x 10^-07^* |
|  | rs34879941 | MODIFIER | 0.581 | *7.941 x 10^-15^* | *0.002* | *4.155 x 10^-05^* | *8.123 x 10^-12^* | *9.199 x 10^-10^* |
|  | rs36038527 | MODIFIER | 0.581 | *2.721 x 10^-14^* | 0.199 | *3.501 x 10^-04^* | *2.817 x 10^-12^* | *2.183 x 10^-11^* |
|  | rs4823173 | MODIFIER | *1.021 x 10^-19^* | 0.096 | *1.341 x 10^-16^* | *7.634 x 10^-10^* | *0.011* | *0.001* |
| *SERPINA6* | rs1042394 | LOW | 0.181 | *0.002* | 0.052 | 0.858 | *1.977 x 10^-06^* | *0.013* |
|  | rs2228542 | LOW | 0.181 | *5.015 x 10^-05^* | *0.033* | 0.858 | *1.977 x 10^-06^* | *0.013* |
|  | rs3748320 | LOW | 0.098 | *0.025* | 0.098 | 0.229 | *5.086 x 10^-05^* | *0.003* |
|  | rs11160168 | MODIFIER | 1 | *1.294 x 10^-13^* | *2.521 x 10^-06^* | 1 | *5.365 x 10^-10^* | *2.620 x 10^-09^* |
|  | rs11160169 | MODIFIER | *4.043 x 10^-30^* | *9.375 x 10^-04^* | *1.656 x 10^-14^* | *7.162 x 10^-34^* | *4.488 x 10^-05^* | *1.069 x 10^-11^* |
|  | rs2273399 | MODIFIER | 0.088 | *5.466 x 10^-04^* | 0.508 | *2.826 x 10^-12^* | 0.181 | *7.587 x 10^-06^* |
|  | rs2281520 | MODIFIER | 0.181 | *5.015 x 10^-05^* | *0.033* | 0.858 | *1.977 x 10^-06^* | *0.013* |
| *SHMT1* | rs142013082 | MODIFIER | *0.002* | *1.101 x 10^-07^* | *0.001* | *1.183 x 10^-05^* | *3.090 x 10^-07^* | *0.001* |
|  | rs2273028 | MODIFIER | *0.013* | *5.816 x 10^-09^* | 0.055 | 0.295 | *0.004* | 0.485 |
|  | rs2273029 | MODIFIER | 0.452 | 0.974 | *0.011* | *0.002* | *0.032* | 0.737 |
|  | rs3866955 | MODIFIER | *1.679 x 10^-36^* | *0.011* | *2.757 x 10^-06^* | *2.438 x 10^-04^* | *1.168 x 10^-08^* | *2.078 x 10^-07^* |

**SUPPLEMENTARY TABLE S2.** Allele frequencies of variants from Table 3, with high or moderate impact and significant in three or more populations.

| **Gene** | **Variant** | **Impact** | **IND-NLLA x LLA** | **IND-NLLA**  **x AFR** | **IND-NLLA**  **x AMR** | **IND-NLLA**  **x EAS** | **IND-NLLA**  **x EUR** | **IND-NLLA**  **x SAS** |
| --- | --- | --- | --- | --- | --- | --- | --- | --- |
| *ABCB1* | rs2032582 | Moderate | *2.32 x 10^-35^*(-) | *8.93 x 10^-51^*(-) | *8.43 x 10^-22^*(-) | *8.37 x 10^-18^*(-) | *2.94 x 10^-22^*(-) | *2.82 x 10^-12^*(-) |
| *ADH1C* | rs1693482 | Moderate | *1.16 x 10^-9^*(+) | *6.51 x 10^-3^*(+) | 0.97(-) | *8.13 x 10^-4^*(+) | *3.07 x 10^-2^*(-) | 0.97(-) |
|  | rs35719513 | Moderate | *1.21 x 10^-6^*(+) | *1.21 x 10^-6^*(+) | *1.41 x 10^-2^*(+) | *1.21 x 10^-6^*(+) | *1.21 x 10^-6^*(+) | *1.21 x 10^-6^*(+) |
| *FGFR4* | rs1966265 | Moderate | 0.13(+) | *1.21 x 10^-20^*(+) | *2.31 x 10^-5^*(+) | 0.26(+) | *3.53 x 10^-9^*(+) | *3.53 x 10^-9^*(+) |
|  | rs351855 | Moderate | *1.54 x 10^-6^*(-) | *4.03 x 10^-4^*(-) | *3.70 x 10^-16^*(-) | *5.58 x 10^-17^*(-) | *1.72 x 10^-10^*(-) | *1.20 x 10^-12^*(-) |
|  | rs376618 | Moderate | 0.33(-) | *2.41 x 10^-10^*(+) | *1.02 x 10^-2^*(+) | 0.33(-) | *1.68 x 10^-6^*(+) | *2.88 x 10^-2^*(+) |
| *GSTA1* | rs1051775 | High | *5.47 x 10^-4^*(-) | 0.26(-) | *5.47 x 10^-4^*(-) | 0.05(-) | *6.63 x 10^-12^*(-) | *7.10 x 10^-9^*(-) |
| *HMMR* | rs299284 | Moderate | *1.84 x 10^-2^*(-) | *3.14 x 10^-2^*(-) | 0.24(-) | 1(+) | *3.22 x 10^-3^*(-) | *1.84 x 10^-2^*(-) |
|  | rs299295 | Moderate | 0.43(-) | *1.19 x 10^-5^*(-) | *3.38 x 10^-2^*(-) | 1(-) | *2.39 x 10^-4^*(-) | *7.39 x 10^-5^*(-) |
| *NOS1* | rs11068428 | Moderate | *2.68 x 10^-15^*(+) | *6.65 x 10^-21^*(+) | *1.05 x 10^-3^*(+) | *2.37 x 10^-7^*(+) | *2.01 x 10^-14^*(+) | *3.71 x 10^-13^*(+) |
| *PNPLA3* | rs2076213 | Moderate | 1(-) | *1.06 x 10^-6^*(+) | *4.55 x 10^-2^*(+) | *4.94 x 10^-8^*(+) | *2.22 x 10^-4^*(+) | *2.22 x 10^-4^*(+) |
|  | rs2294918 | Moderate | *3.28 x 10^-3^*(+) | 0.11(+) | *5.41 x 10^-3^*(+) | *2.24 x 10^-2^*(+) | *7.29 x 10^-10^*(+) | *4.07 x 10^-5^*(+) |
|  | rs738409 | Moderate | 0.44(+) | *2.69 x 10^-16^*(+) | 0.24(+) | *4.07 x 10^-5^*(+) | *7.27 x 10^-11^*(+) | *2.78 x 10^-11^*(+) |
| *SERPINA6* | rs2228541 | Moderate | 0.295(+) | *3.59 x 10^-20^*(+) | *7.18 x 10^-6^*(+) | 0.87(+) | *1.90 x 10^-17^*(+) | *3.49 x 10^-9^*(+) |
| *SHMT1* | rs1979277 | Moderate | *7.68 x 10^-3^*(-) | *1.34 x 10^-3^*(-) | *2.50 x 10^-2^*(-) | 0.11(+) | *5.05 x 10^-3^*(-) | 1(-) |

*Symbols (+) and (-) indicate whether the variant frequency in the IND-NLLA population is higher or lower compared to other populations. For detailed frequency values, see Supplementary Table S2.*

**SUPPLEMENTARY TABLE S3.** Information about genes, gene function and relation with *glucocorticoids*

| ***GENE*** | ***GENE FUNCTION*** | ***RELATION WITH GLUCOCORTICOIDS*** | ***REFERENCE*** |
| --- | --- | --- | --- |
| *ABCB1* | Encodes a membrane-associated protein that functions as an ATP-dependent drug efflux pump with broad substrate specificity, involved in multidrug resistance. | Associated with corticosteroid-induced toxicity, and polymorphisms affect response to prednisone therapy. | www.ncbi.nlm.nih.gov/gene; www.pharmgkb.org/clinicalAnnotation; Article:15110230 |
| *ACP1* | Regulates signaling processes such as cell growth, proliferation, and differentiation by modulating protein phosphorylation and activity of phosphorylated monoesters. | Genotype TT linked to increased osteonecrosis risk in corticosteroid-treated pediatric leukemia patients. | www.ncbi.nlm.nih.gov/gene; www.pharmgkb.org/clinicalAnnotation; dx.doi.org/10.1182%2Fblood-2010-10-311969 |
| *ADH1C* | Encodes a class I alcohol dehydrogenase that metabolizes ethanol, retinol, and other substrates, playing a role in lipid metabolism and detoxification. | Dexamethasone treatment increases total ADH1 mRNA levels, enhancing transcription. | www.ncbi.nlm.nih.gov/gene; 10.1073/pnas.85.3.767 |
| *BCL2L11* | Induced by nerve growth factor and involved in apoptosis in neurons and lymphocytes, with expression in lymph nodes. | Variants associated with corticosteroid-induced osteonecrosis in pediatric leukemia patients. | www.ncbi.nlm.nih.gov/gene; www.pharmgkb.org/gene/PA25305/clinicalAnnotation |
| *BMP7* | Encodes a TGF-beta ligand that regulates bone, kidney, and brown adipose tissue development by activating SMAD family transcription factors. | Variant associated with osteonecrosis risk in dexamethasone and prednisone-treated pediatric leukemia patients. | www.ncbi.nlm.nih.gov/gene; www.pharmgkb.org/clinicalAnnotation/1448099112 |
| *CTNNB1* | Part of adherens junction complexes, regulating cell growth, adhesion, and actin cytoskeleton anchoring, crucial for maintaining epithelial cell layers. | Variant associated with response to dexamethasone in multiple myeloma patients. | www.ncbi.nlm.nih.gov/gene; www.pharmgkb.org/variantAnnotation/1447520580 |
| *CXCL12* | Acts as a ligand for the G-protein coupled receptor CXCR4, playing roles in embryogenesis, immune response, inflammation, and tumor growth. | Variant associated with prednisone response in chronic lymphocytic leukemia patients. | www.ncbi.nlm.nih.gov/gene; www.pharmgkb.org/clinicalAnnotation/1448099828 |
| *DROSHA* | Encodes a ribonuclease enzyme involved in processing double-stranded RNA and microRNA molecules, crucial for regulating gene expression. | Variant associated with prednisone-induced toxicity in pediatric leukemia patients. | www.ncbi.nlm.nih.gov/gene; www.pharmgkb.org/clinicalAnnotation/1447990283 |
| *FGFR4* | Encodes a tyrosine kinase receptor involved in regulating cell proliferation, migration, metabolism, and homeostasis of phosphate, lipids, and glucose. | Functional Role in Metabolism and Homeostasis. No direct glucocorticoid relation mentioned. | www.ncbi.nlm.nih.gov/gene/2264 |
| *FOLH1* | Encodes a transmembrane glycoprotein that acts as a glutamate carboxypeptidase on substrates like folate and neuropeptides, expressed in several tissues. | Functional Role in Metabolism and Homeostasis. No direct glucocorticoid relation mentioned. | www.ncbi.nlm.nih.gov/gene/2346 |
| *GATA3* | Encodes a transcription factor critical for T-cell development and endothelial cell regulation, belonging to the GATA family of zinc-finger proteins. | Variant associated with worse glucocorticoid treatment response in pediatric leukemia patients. | www.ncbi.nlm.nih.gov/gene/2625; www.pharmgkb.org/clinicalAnnotation/1184512128 |
| *GSTA1* | Encodes a member of the glutathione S-transferase superfamily, critical for detoxifying harmful compounds through conjugation with glutathione. | Expression regulated by glucocorticoid receptor binding, enhancing detoxifying activity in response to dexamethasone. | www.ncbi.nlm.nih.gov/gene/2944; doi.org/10.1016/j.tox.2017.05.002 |
| *HMMR* | Encodes a receptor for hyaluronic acid, playing roles in cell motility, transformation, and regulation of adipogenesis and ERK activity. | Established Roles in Disease Pathways. No direct glucocorticoid relation mentioned. | www.ncbi.nlm.nih.gov/gene/3161 |
| *NOS1* | Encodes a nitric oxide synthase that synthesizes nitric oxide, involved in neurotransmission, smooth muscle regulation, and immune activities. | Established Roles in Disease Pathways. No direct glucocorticoid relation mentioned. | www.ncbi.nlm.nih.gov/gene/4842 |
| *NR3C1* | Encodes the glucocorticoid receptor, functioning as a transcription factor involved in inflammatory responses, cellular proliferation, and differentiation. | Encodes the glucocorticoid receptor itself, mediating glucocorticoid responses and resistance. | www.ncbi.nlm.nih.gov/gene/2908 |
| *PNPLA3* | Encodes a triacylglycerol lipase that mediates lipid breakdown, regulating energy usage and storage, and is expressed in various tissues. | Expression regulated by insulin signaling and other pathways, with potential involvement in lipid regulation. | www.ncbi.nlm.nih.gov/gene/80339; dx.doi.org/10.1080/14728222.2021.2018418 |
| *SERPINA6* | Encodes a corticosteroid-binding globulin, with roles in binding and transporting corticosteroids like prednisone and prednisolone in plasma. | Binds and transports corticosteroids like prednisone and prednisolone in plasma. | www.pharmgkb.org/pathway/PA166273601 |
| *SHMT1* | Encodes a cytosolic serine hydroxymethyltransferase, essential for providing one-carbon units for synthesizing methionine, thymidylate, and purines. | Functional Role in One-Carbon Metabolism. No direct glucocorticoid relation mentioned. | www.ncbi.nlm.nih.gov/gene/6470 |
